# Thrombosis and Aspirin: Clinical Aspect, Aspirin in Cardiology, Aspirin in Neurology, and Pharmacology of Aspirin

**DOI:** 10.1155/2012/626289

**Published:** 2012-10-21

**Authors:** Christian Doutremepuich

**Affiliations:** Laboratoire d'Hématologie, Université Victor Segalen Bordeaux 2, 146 Rue LéoSaignat, 33076 Bordeaux Cedex, France

Acetyl salicylic acid or aspirin is one of the most famous drugs in the world. Aspirin has been increasingly used for prevention of cardiovascular events and is, particularly in recent decades, the most used nonsteroidal anti-inflammatory drug. 

Thus, it was necessary to have several contributions to precise the interest and the side effects of aspirin in cardiology, pharmacology, and neurology.

A general review on aspirin pharmacology was made by Espinosa et al. (Western University of Health Sciences, Pomona, CA, USA). This review article helps to understand the role of platelets in primary hemostasis and atherothrombosis, the use of aspirin in the prevention, and treatment of cardiovascular diseases.

In the review article by B. Rocca and G. Petrucci (Catholic University School of Medicine, Rome, Italy), they have detailed the phenomenon of variability in the responsiveness to low-dose aspirin and reported the explanations of this real problem in patients.

The clinical use of aspirin in treatment and prevention of cardiovascular diseases was the subject of the work of Y. Dai and J. Ge (Fudan University, Shanghai, China). In this paper, the authors review the different clinical situations when aspirin is administered.

The use of aspirin for prevention of thrombosis was clearly discussed by G. H. R. Rao and J. Fareed (University of Minnesota, Minneapolis, MN and Loyola University Medical Center, Maywood, IL, USA).

The special work on the role of dermcidin isoform 2 by Ghosh et al. (Calcutta, India) completes the two clinical papers.

The work of Lösche et al. (Jena University Hospital, Jena, Germany) on the problem of the reduction of mortality in critically ill patients also demonstrates the effectiveness of antiplatelet drugs to prevent organ failure.

The three experimental studies from the laboratory of Doutremepuich concern the paradoxical action of aspirin used at different dosages. These different effects can explain the thrombosis observed in clinical practice after aspirin discontinuation. The question could be also: what is the effect of a drug at ultralow dose?

In conclusion, this special issue can represent a “state of art” on aspirin and thrombosis with new hypotheses of work.

